# Amino Acid Cross-Linked Graphene Oxide Membranes for Metal Ions Permeation, Insertion and Antibacterial Properties

**DOI:** 10.3390/membranes10100296

**Published:** 2020-10-21

**Authors:** Lijuan Qian, Haijing Wang, Jingyi Yang, Xiaolei Chen, Xue Chang, Yu Nan, Zhuanyan He, Peizhuo Hu, Wangsuo Wu, Tonghuan Liu

**Affiliations:** 1 School of Nuclear Science and Technology, Lanzhou University, Lanzhou 730000, China; qianlj@lzu.edu.cn (L.Q.); wanghj16@lzu.edu.cn (H.W.); yangjy19@lzu.edu.cn (J.Y.); sophia93@foxmail.com (X.C.); changx20@lzu.edu.cn (X.C.); nany18@lzu.edu.cn (Y.N.); hezhy14@lzu.edu.cn (Z.H.); wuws@lzu.edu.cn (W.W.); 2 Key Laboratory of Special Function Materials and Structure Design, Ministry of Education, Lanzhou 730000, China

**Keywords:** graphene oxide membrane, cross-linking, amino acid, metal ions, rejection

## Abstract

Graphene oxide (GO) and its composite membranes have exhibited great potential for application in water purification and desalination. This article reports that a novel graphene oxide membrane (GOM) of ~5 µm thickness was fabricated onto a nylon membrane by vacuum filtration and cross-linked by amino acids (L-alanine, L-phenylalanine, and serine). The GOM cross-linked by amino acids (GOM-A) exhibits excellent stability, high water flux, and high rejection to metal ions. The rejection coefficients to alkali and alkaline earth metal ions through GOM-A were over 94% and 96%, respectively. The rejection coefficients decreased with an increasing H^+^ concentration. Metal ions (K^+^, Ca^2+^, and Fe^3+^) can be inserted into GOM-A layers, which enlarges the interlayer spacing of GOM-A and neutralizes the electronegativity of the membrane, resulting in the decease in the rejection coefficients to metal ions. Meanwhile, GOM-A showed quite high antibacterial efficiency against E. coli. With the excellent performance as described above, GOM-A could be used to purify and desalt water.

## 1. Introduction

Nowadays, all living things on the earth are being threatened greatly by the shortage and pollution of water because of the fast population growth and environmental pollution caused by industry development [1,2], so water treatment and purification are of great significance to the development of society and economy. Generally, wastewater treatment methods are composed of precipitation, microbial decomposition, physical adsorption, membrane separation, etc. [3]. Membrane technology has been widely used in seawater desalination and wastewater treatment for its high efficiency, simple operation, lack of phase-change, and limited chemical requirements [4]. However, it is a great challenge to fabricate membranes with high stability, high flux and good antibacterial ability.

Graphene and its derivatives, graphene oxide, are two-dimensional materials with excellent chemical and thermal stability [5]. Recently, it was reported that monolayer nanoporous graphene can be used to separate ions with high efficiency and selectivity [6,7], but the method is difficult to apply in practical applications due to the high cost and complex process. Therefore, a multilayer graphene oxide membrane (GOM) with controllable layer spacing has received extensive attention due to the simple, fast, and low-cost preparation [8,9]. Nanochannels stacked between layers of GOMs make it possible for molecules or ions to penetrate through the membranes. Water can immerse into Graphene oxide (GO) sheets and form 2–3 layers of water molecule intercalation when permeating through the GO layer. This will cause membrane swelling and an interlayer spacing enlargement of GOM, leading to a decrease in the stability of GOM. [10]. The cross-linked modification of GOMs helps to form hybrid structures, which can increase membrane stability greatly and decrease swelling properties. Common cross-linking methods are chemical cross-linking [11,12,13] and polyvalent metal ion cross-linking [14,15]. Diamine [16], tannic acid [12], polyethyleneimine [17], and dopamine [18,19] are often used to cross-link with GO. After cross-linking, the d-spacing of GO lamellas could be fixed by the length of cross-linking agents [20]. Consequently, the stability of the GOM in the solution could be enhanced after cross-linking, which enables the wide application of enhanced membranes in the researches of metal ions and organic matter permeation. However, whether cross-linked GOMs retain the metal ions in GO lamellas and whether the retained ions affect the permeating ions still require further study.

Three kinds of amino acid, with different carbon numbers and hydrophilicities, were used as cross-linking agents to modify GOMs in this work. The carboxyl and amino groups of the amino acid may be bonded to the carboxyl and hydroxyl groups on GO so that GO layers are fixed by the carbon chain of the amino acid forming stable complexes. L-alanine, L-phenylalanine, and serine were selected to modify GOMs (named GO-Ala, GO-PHE, and GO-Ser, respectively) in this study to explore the influence on the properties of membranes. Their stability, water flux, and rejection coefficients to alkali metal and alkaline earth metal ions were evaluated. GO-PHE, inserted by metal ions (K^+^, Ca^2+^, Fe^3+^, named GO-PHE-K, GO-PHE-Ca, GO-PHE-Fe, respectively), were characterized by XRD and XPS to determine the content and effect of metal ions. The permeation of sodium ions through the membranes (GO-PHE-K, GO-PHE-Ca, GO-PHE-Fe) was investigated to identify the effect of interlaminar metal ions. Furthermore, the antibacterial activity of the GOM cross-linked by amino acids was also investigated.

## 2. Experimentals

### 2.1. Regents and Materials

Graphite powder (≧98.0%) was purchased from Aladdin Industrial Inc. (Shanghai, China). Sodium nitrate (NaNO_3_), Potassium nitrate (KNO_3_), Calcium nitrate (Ca(NO_3_)_2_), and Magnesium Nitrate (Mg(NO_3_)_2_) were purchased from Tianjin Guangfu Technology Development Co. LTD, (Tianjin, China). Potassium permanganate (KMnO_4_) was purchased from saan chemical technology Co. LTD, (Shanghai, China). Hydrogen peroxide (H_2_O_2_) (30%) was purchased from Xilong Chemical Co. LTD, (Guangzhou, China). L-alanine, L-phenylalanine and serine (≥99.0%) were obtained from Shanghai Zhongqin Chemical Reagent Co. LTD, (Shanghai, China). A nylon membrane with a pore size of 0.22 µm was obtained from Taoyuan Medical Chemical Instrument Factory (Haining, China). Nitric acid, hydrochloric acid and sulfuric acid was obtained from the Chengdu Kelon Chemical Reagent Factory (Chengdu, China).

Deionized water was used by in all experiments. All reagents were analytical grade and used as received without further purification.

### 2.2. Preparation and Characterizations of GOMs

The modified Hummers method was used to prepare a GO suspension (see Supplementary Information 1, Preparation of GO) [21]. The powders of L-alanine, L-phenylalanine and serine were added to a 400-ppm GO suspension and the concentration of amino acid was 0.1 mol/L, respectively. After sonicating for 5 min, the mixture (2 mL) was filtered by vacuum filtration onto a nylon membrane to prepare the composite GOMs (the diameter of the membrane was 2.5 cm). Concentrated hydrochloric acid was dropped on the membrane surface to catalyze the reaction. Then the composite membrane was placed in an oven and heated at 80 °C for 1 h. After that, the membrane was immersed in anhydrous methanol for 1 h, then washed with distilled water and dried naturally. The product membranes are named GO-Ala, GO-PHE, and GO-Ser, respectively, and the thicknesses of the membranes are about 5 µm. The stability of the membranes was studied and listed in Supplementary Information 3.

GO-Ala, GO-PHE, and GO-Ser were characterized by FT-IR, X-ray diffraction (XRD), SEM, X-ray photoelectron spectroscopy (XPS), Atomic force microscopy (AFM), and a contact angle measuring instrument. FT-IR spectroscopy (Nicolet, Madison, WI, USA) was registered in the range of 500–4000 cm^−1^, using a nominal resolution of 4 cm^−1^ and 32 scans per spectrum. Powder X-ray diffraction (XRD) measurements were performed on an X’pert Pro MRD PANalytical diffractometer with a CuKα anticathode (λ_Kα1_ = 0.1540598 nm, λ_Kα2_ = 0.1544426 nm) and the current of the lamp is 40 mA. X-ray patterns were collected in the 4–40° 2θ range with steps of 0.02°; the acquisition time was 10 s per step. The spectrogram was processed by flattening the baseline. Scanning electron microscopy (SEM) was performed with a field emission scanning electron microscope MIRA3. The microscopic features of these materials were taken with an accelerating voltage of 15 kV (Tescan, Bmo, South Moravia, Czech Republic). The samples were sputtered with thin layers of Au. The cross-section of samples for analysis was prepared by cutting the film with a blade.

The membrane surface composition was analyzed by X-ray photoelectron spectroscopy (XPS) (Axis Ultra DLD, Kratos, UK) with a monochromatic Al Ka source at a power of 180 W (15 kV × 12 mA). When the instrument is operating, the level of vacuum is ~2 × 10^−9^ mbar, and the take-off angle between the X-ray and the sample surface normal is 45 degrees. The surface pollution C1s (284.8 eV) was used as the standard for energy correction and the irradiated surface area was 700 × 300 μm. For comparison, a layered GOM was prepared by spincoating a dilute GO solution (1.0 mg mL^−1^) on the nylon substrate with the same method as that for GOM-A membrane fabrication.

Atomic force microscopy (AFM) images were recorded using an Icon (Burker Corperation, Santa Barbara, CA, USA). The scanning probe microscope (SPM) was equipped with a silicon tip on nitride lever (Scanasyst-Air) with a force constant k = 0.4 N/m and a curvature radius r = 10 nm. The AFM was operated in non-contact mode at a resonance frequency of f = 86.5 kHz. Image scanning was performed with a speed of 1 Hz, which corresponds to one line per second.

Contact angle measurement was conducted by Kruss DSA100 (Xinrui, Xiamen, China). Before conducting measurements, samples were dried overnight at room temperature. A drop of deionized water (5 μL) was dropped onto the smooth, flat membrane surface from a microsyringe with a stainless-steel needle. The instrument software was set to record data at 60 FPS for 5 s. To reduce error, a reliable contact angle value was obtained by averaging 5 measurements obtained from different membrane surfaces.

### 2.3. Measurement of Water Fluxes, and Test of Stability and Tensile Properties

Schematic diagram of the water flux measurement device is shown in Figure S1 [22]. The permeate device consisted of two osmosis tanks, isolated by the membrane. There was one overflow on the upper part and one inlet on the lower part of each tank. The GO-Ala (GO-PHE or GO-Ser) membrane was fixed between the middle of two tanks. The green and blue beakers were separately filled with salt solution and deionied water. By turning on the peristaltic pumps, the solution in the beakers flowed into the osmotic tanks from the bottom inlet. When the solution in the osmosis tanks reach the overflow aperture, the solution overflowed to the beaker. The weight of the salt solution was recorded on the electrical scale every 5 min when the liquid cycle was stable. According to Formula (1), water fluxes of GOM, GO-Ala, GO-PHE, and GO-Ser can be obtained:

*J* = *V*/*A* × *t* (1)

where *J* is the membrane water flux (L/m^2^·h), *V* is the volume change (L) of the salt solution, *t* is the osmotic time (h), and *A* is the effective area of the membrane (m^2^), which is 2.54 × 10^−4^ m^2^ in this work.

### 2.4. Stability and Tensile Properties of GOMs

GOM, GO-Ala, GO-PHE, and GO-Ser membranes were soaked in deionized water for ≈24 h. Then these membranes were treated by ultrasonic apparatus for verifying the stability of membranes. The tensile properties of the composite membranes at a stretching speed of 50 m/min were examined using an electronic universal testing machine (WDW-200, Bairoe, Shanghai, China) controlled by a microcomputer.

### 2.5. Permeation Experiment

The sketch of the permeate device is shown in Figure S1—two tanks isolated by an osmosis membrane (with 2 cm diameter) without overflow and inlet. One of the tanks (named the feed tank) was filled with 0.05 mol/L metal ion solution, another tank (named the permeate tank) used nitric acid as draw solution. The membranes were fixed between feed and permeate tanks by clamping the device. A total of 50 mL of metal ion solution and the nitric acid solution was injected into the tanks, respectively. Magnetic stirrings were used in both tanks to promote the permeation. After 12 h, the two tanks were sampled, and the concentrations of metal ions were measured by an atomic absorption spectrometer (AAnalyst 700, PerkinElmer Company, Waltham, MA, USA).

The rejection coefficient of metal ions was obtained by the following Equation (2):

R% = (1 − C_f_/C_p_) × 100% (2)

where R is the rejection coefficient, C_f_ and C_p_ (mg/L) are the final metal ion concentrations in the permeate tank and initial metal ion concentration in the feed tank, respectively.

The metal ion permeation flux was obtained by Equation (3):

*J_p_* = *c* × *V*/*A* × *t* (3)

where *J*_p_ is the metal ion permeation flux (mmol/m^2^·h), *c* is the metal ion concentration in the permeate tank.

### 2.6. GO-PHE Membrane Insertion Experiment

GO-PHE membranes were immersed in 0.05 mol·L^−1^ of KNO_3_, Ca(NO_3_)_2_, and Fe(NO_3_)_3_ solutions for ~12 h, respectively. The membranes impregnated with salt solutions were rinsed several times with deionized water and dried naturally. The rejection coefficient of Na^+^ by the membranes was studied, and the experiment scheme was the same as the scheme of the permeation experiment.

### 2.7. Antibacterial Ability Measurement

The antibacterial ability of GOM, GO-Ala, GO-PHE, and GO-Ser was investigated by inoculating *E. coli* onto the membranes [23]. After incubating *E. coli* with Luria-Bertani (LB) media at 37 °C overnight, the suspension was then diluted to be dispersed evenly onto 50-µm thick GOMs for 2 h at 37 °C. The bacteria solution was washed out with 9 mL of saline solution (0.9% NaCl). In total, 0.1 mL of the washing solution was inoculated onto an LB agar plate evenly and incubated at 37 °C for 24 h. The control group was with the same condition but without GOMs. After incubation, the colonies on the agar plate were counted, and the antibacterial rate was calculated by Equation (4)

antibacterial rate = (B − A)/B × 100% (4)

where A is the number of visible bacterial colonies with the GOM of the experimental group, and B is the number of visible bacterial colonies on the control plate. The final data are the averages of the data of three parallel experiments.

## 3. Results and Discussion

### 3.1. Characterization of GO-Ala, GO-PHE, GO-Ser

**Figure 1:**
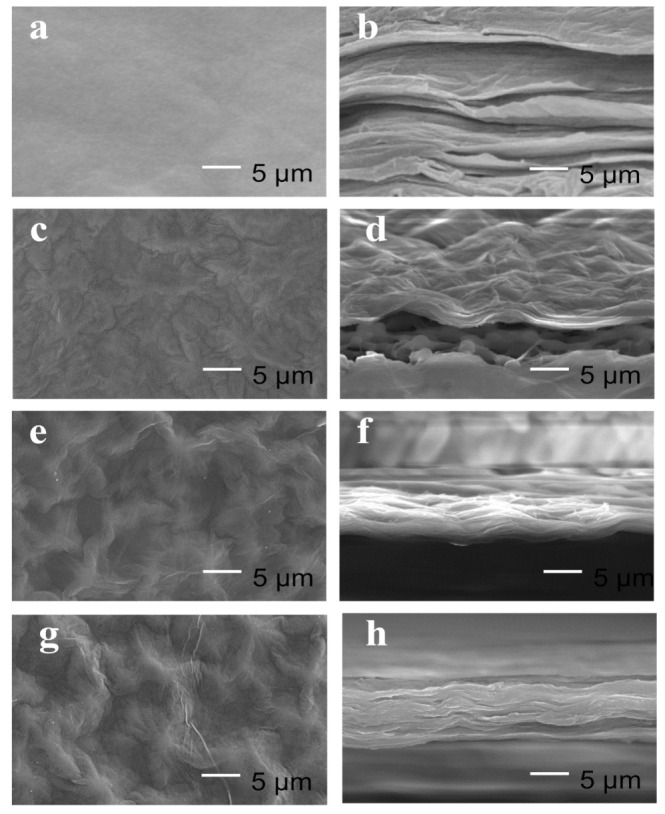
The Scanning electron microscopy (SEM) images of surface graphene oxide membrane (GOM) (**a**), graphene oxide (GO)-L-alanine (Ala) (**c**), GO-serine (Ser) (**e**), GO- L-phenylalanine (PHE) (**g**) and cross-section of GOM (**b**), GO-Ala (**d**), GO-Ser (**f**), GO-PHE (**h**).

It can be observed from SEM images (Figure 1a,b) that the surface of GOMs are smooth with typical wrinkle morphologies and lamellar structures, which is consistent with GOMs in the literature reported [20,24]. GO-Ala, GO-Ser, and GO-PHE membranes possess a wrinkled surface topography (Figure 1c,e,g) and a well-layered lamellar structure (Figure 1d,f,h) similar to the surface of GOMs. The cross-sections of GO-Ala, GO-Ser, and GO-PHE membranes are more orderly and compact than GOMs (see Figure 1b,d,f,h).

**Figure 2:**
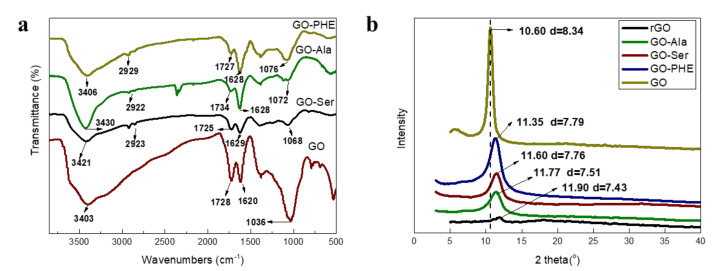
(**a**) FT-IR spectra of GO, GO-Ala, GO-Ser, GO-PHE, and (**b**) X-ray diffraction (XRD) spectra of GO, rGO, GO-Ala, GO-PHE, GO-Ser.

The FT-IR spectra in Figure 2a show that GO has three characteristic peaks at ~3403, ~1728, and ~1036 cm^−1^, assigned to OH, C=O, and C-O respectively [20]. After modifying with amino acids, the intensity of the peaks at 1036 cm^−1^ (C-O) decreased, probably due to the cross-linking reaction of NH_2_ with epoxy groups [25]. GOM-Ser is observed one new absorption peak at wavenumbers of 2351 cm^−1^ and represents carbon dioxide because amine easily absorbs CO_2_ [20]. GO-Ala and GO-PHE have a weak absorption peak at 2351 cm^−1^, perhaps because of a low amount of amine. The O-H and N-H stretching vibration peaks red-shifted at ~3420 cm^−1^, probably indicating the covalent functionalization of GO by amino acid molecules.

From the X-ray scattering data (Figure 2b), the average d-spacing value of GO is derived from Bragg’s law (5) [26]:

(5) $$d=\frac{\lambda }{2\sin\theta }$$

where d is the layer spacing, *λ* is the X-ray wavelength of 1.54 nm, and *θ* is the diffraction angle. The interlayer distances can be obtained from the peak diffraction angle. According to our experimental work, the interlayer distances of the amino acid modified composite membranes are ~7.51 (GO-Ala), ~7.79 (GO-PHE), and ~7.76 Å (GO-Ser), respectively. By comparison, the d-spacing of GO-PHE is slightly larger than that of GO-Ala and GO-Ser for the benzene ring inserted into GO layers. Similarly, Hung et al. [27] reported that the interlayer spacing of p–phenylenediamine cross-linked GO composite membranes was a little larger than the membranes modified with ethylenediamine and butanediamine. The interlayer spacings of GO and rGO (heated for ~1 h) are 8.34 nm and 7.43 Å (Figure 2b), respectively. The rGO treated thermally showed a gradual decrease in d-spacing, for the removal of oxygen-containing groups from GO [28,29]. The interlayer spacings of GO-Ala, GO-PHE, GO-Ser are smaller than that of GO because some oxygen groups have decomposed after one hour of heat treatment.

**Figure 3:**
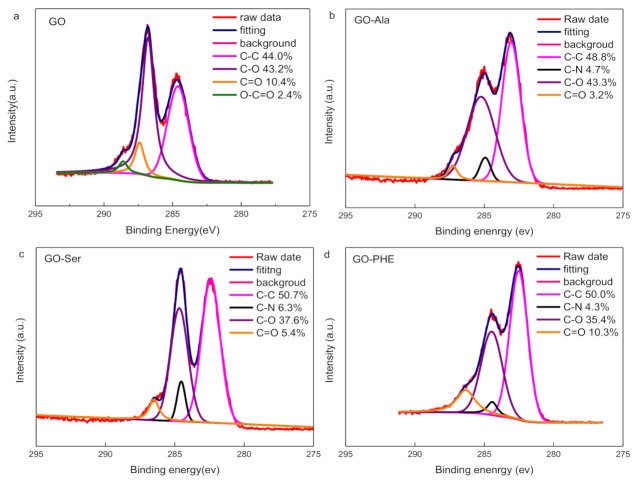
X-ray photoelectron spectroscopy (XPS) C1s spectra of GOM (**a**), GO-Ala (**b**), GO-Ser (**c**), and GO-PHE (**d**).

**Figure 4:**
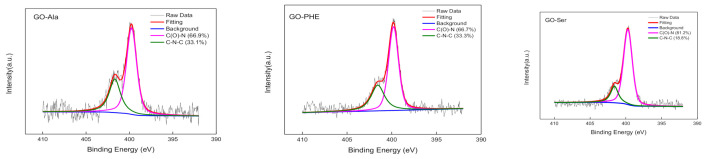
XPS N1s spectra of GO-Ala, GO-PHE, and GO-Ser.

XPS results in Figure 3, Figure 4 and Figure S2 show that GO, GO-Ala, GO-Ser, and GO-PHE exhibit C 1s, O 1s, and N 1s peaks, while GO has C 1s and O 1s peaks only, indicating that GO-Ala, GO-Ser, and GO-PHE contain nitrogen while GO does not. The detailed information of C ls and N 1s spectra of GOMs are shown in Figure 3 and Figure 4, respectively; the peaks are fitted by the linear combination of a Gaussian function and Lorentzian function. GO has four peaks at binding energies of 284.5, 286.7, 288.2, and 289.4 eV, which are consistent with C-C, C-O, C=O, and O-C=O, respectively [30]. The C ls spectra of GO-Ala, GO-Ser, and GO-PHE all show a new peak at 286.2 eV, which is attributed to C-N. Amino acid-cross-linked GO forms C-N covalent bonds, whose percentage composition is about 4%~6%. GO-Ser has the highest amount of C-N covalent bonds (Figure 3) and N (Figure S2) in GOM-A, and appears to have an absorption peak at 2351 cm^−1^ in FT-IR. The total amount of C-O, and C=O in GO-Ala, GO-Ser, and GO-PHE membranes is about 42%~46% while the total amount of C-O, C=O, and O-C=O in GO is about 56%. The cross-linking of amino acids and heat treatment result in the decrease in oxygen content in GO-Ala, GO-Ser, and GO-PHE membranes. From Figure 4, the N ls spectra of GO-Ala, GO-Ser, and GO-PHE shows two peaks at binding energies of 399.7 and 400.3 eV, which are consistent with N-C=O and C-N-C, respectively [31]. XPS spectra present the formation of amide groups in GO-Ala, GO-Ser, and GO-PHE membranes.

**Figure 5:**
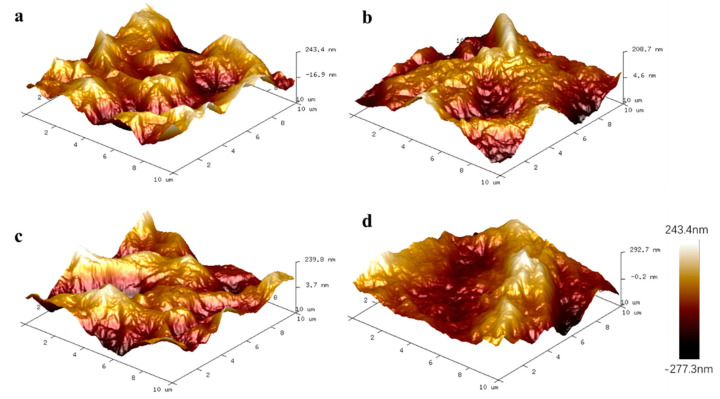
The Atomic force microscopy (AFM) images of surface GOM (**a**), GO-Ala (**b**), GO-Ser (**c**), and GO-PHE (**d**).

The detailed surface morphological profile of the GOMs was further characterized by AFM (Figure 5). In these images, the bright area presents the high point of the membrane surface and the dark regions indicate the valley of the membrane surface distributed throughout the plane. The maximum surface roughness (R_max_), root average arithmetic roughness (Ra), and root peak-to-valley distance (Rpv) of the membrane surface are listed in Table S1. This observation from the surface morphological profile agrees with the SEM results. The surface roughness of the GOM and GOM-A is almost the same. It can be concluded that cross-linking by amino acids did not affect surface roughness.

**Figure 6:**
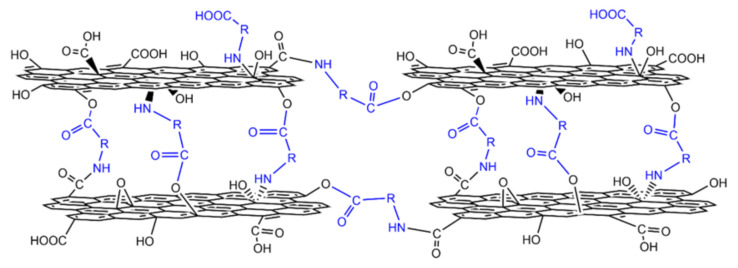
Microstructure model of GOM cross-linked with amino acids.

Schematic view for the possible structure of GOM cross-linked by amino acids is illustrated in Figure 6. The carboxyl and amino groups of the amino acid can be bonded to hydroxy, carboxyl, and epoxyl groups on GO, so that GO layers can be fixed by the carbon chain of the amino acid with formed stable complexes.

### 3.2. Contact Angle, Water Flux, Stability and Tensile Properties of GOM, GO-Ala, GO-PHE, and GO-Ser

We explored the hydrophilicity of GO, GO-Ala, GO-PHE, GO-Ser by measuring contact angles. When the contact angle α < 90°, it means that the surface of the solid sample shows good hydrophilicity. A smaller contact angle indicates better hydrophilicity [32]. The contact angle of GO, GO-Ala, GO-PHE, and GO-Ser was 52°, 44.4°, 53.8°, and 53.2°, respectively (Table 1), exhibiting their good hydrophilicity [33].

The water flux of the membranes was measured by a self-made device (Figure S1). The volumes of water through the membrane were obtained from water mass change Δm, which were 0.15, 0.10, 0.14 g, and 0.25 g per 5 min for GOM, GO-Ala, GO-Ser, and GO-PHE, respectively. According to Formula (1), the water fluxes for GOM, GO-Ala, GO-Ser, and GO-PHE were 6.07, 4.72, 6.61, and 11.81 L/m^2^·h, respectively (Table 1). The water fluxes of GOM-A gradually increase with the layer spacing. The functional groups of GOM-A membranes may reduce interactions between the membranes and water molecules to facilitate water molecules’ permeation although GO-Ser and GO-PHE have small layer spacing compared to GOMs. GO-PHE prepared about 5-µm thick has a relatively large water flux in this study compared with the references [11,12,34].

The stability of GOMs, GO-Ala, GO-Ser, and GO-PHE was studied by immersion in deionized water. These membranes remained intact after 24 h in the solutions and were ultrasonicated. The stability data are listed in Table 1. GO-Ala, GO-PHE, and GO-Ser membranes in water were broken into pieces in 30–50 s under sonication, while GOMs were only maintained for 14 s, which means that the modification elongates the lifetime of the membrane 2–3 times.

The tensile strength of the membranes was tested; the tensile strengths of GOM, GO-Ala, GO- PHE, and GO- Ser were 27.2, 26.4, 25.8, and 27.3 MPa, respectively (Table 1). Thus, the composite membranes all possess good tensile strength [35].

### 3.3. Rejection Coefficients of GO-Ala, GO-PHE, and GO-Ser

**Figure 7:**
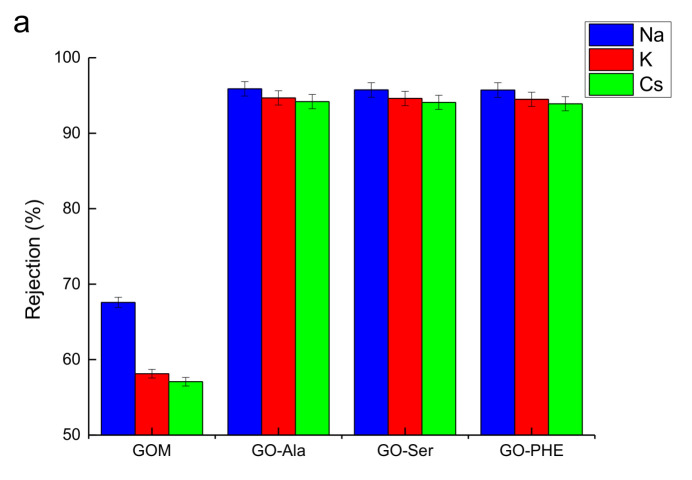

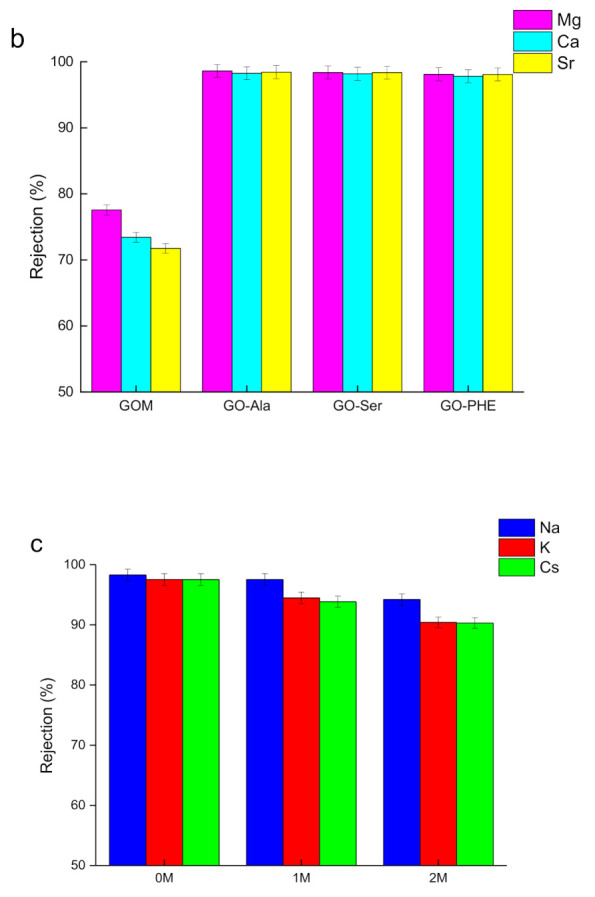
Rejection coefficient of GO-Ala, GO-PHE, and GO-Ser to alkali metal ion (**a**,**b**) and effect of the acidity on to alkali metal ion permeation through GO-PHE (**c**). [M] = 0.05 mol/L (**a**–**c**), [HNO_3_] = 1.0 mol/L (**a**,**b**).

The rejection performance (or permeate flux) of metal ions through GOMs, GO-Ala, GO-PHE, and GO-Ser was investigated (Figure 7, Table S2). As shown in Figure 7a, the rejection coefficients of alkali metal ions Na^+^, K^+^, Cs^+^ through GO-Ala, GO-PHE, and GO-Ser are all greater than 93%, and follow the order Na^+^ > K^+^ > Cs^+^ for their hydrated ionic radii are 3.58, 3.31, 3.29 Å (R_Na_ > R_K_ > R_Cs_) [36], respectively. The results follow that the ion rejection coefficients decrease with the hydrated ionic radius [24]. The rejection coefficients of GO-Ala, GO-PHE, and GO-Ser to the same alkali metal ion were almost the same for there is little difference in the interlayer distance between the GOM-A.

Figure 7b shows the rejection coefficient to divalent ions Mg^2+^, Ca^2+^, Sr^2+^ through GO-Ala, GO-PHE, and GO-Ser. These metal ions have the same charge number, and all the hydrated ionic radii are in the range of 4.01~4.30 Å [36]. The rejection coefficients of divalent ions through the membranes are all over 96%. The interlayer spacing of GO-Ala, GO-PHE, and GO-Ser detected by XRD is 7.51, 7.79, and 7.76 Å, respectively—all smaller than the hydrated ionic diameter of the divalent ions. It can be concluded that the permeation of divalent ions through the cross-linked membranes is very difficult and perhaps only bare ions pass through the membranes [16].

The rejection coefficients of Na^+^, K^+^, Cs^+^ through GOMs are over 55%, and Mg^2+^, Ca^2+^, Sr^2+^ are over 70% (Figure 7a,b). The rejection coefficients of GOMs are much lower than GOM-A. Compared to GOM-A, GOMs swell in water and the d-spacing of GOMs is enlarged when water molecules enter into GO lamellas leading to metal ions’ low rejection coefficients [10].

According to Table S2, the permeation flux of alkali metal ions is about 0.038–0.045 mmol/m^2^·h, and the permeation flux of alkali earth metal ions is about 0.011–0.022 mmol/m^2^·h through GOM-A. Lim [12] reported that ion permeation fluxes through the cross-linked TA-GO membrane for 2 h were about 18 (K^+^) and 3 mmol m^2^·h (Ca^2+^) when the initial concentration of aqueous MgCl_2_, KCl, CaCl_2,_ and NiCl_2_ solutions was 0.05 mol/L (the same as this experiment). Jia [16] prepared a series of GO membranes cross-linked by dicarboxylic acid and diamine. The permeation fluxes of Na^+^ and K^+^ were 5–85 mmol/m^2^·h. Joshi [10] found that the permeate flux of Mg^2+^ was 500 mmol/m^2^·h when permeation through a 5-mm thick GO membrane from the feed compartment with a 0.2 M solution of MgCl_2_. Notably, the ion permeation fluxes of GOM-A membranes in this experiment are much lower than those of GO-based membranes reported previously.

GO-PHE was chosen to study the effect of H^+^ concentration (in the permeate tank). Figure 7c shows the rejection coefficients of Na^+^, K^+^, Cs^+^ through GO-PHE when the draw solution is deionized water or 1 or 2 mol/L HNO_3_ in the permeate tank. It is obvious that the rejection coefficients through GO-PHE follow the order Na^+^ > K^+^ > Cs^+^—a higher acidity leads to lower rejection. H^+^ ions diffuse from the permeate tank to the feed tank with metal ions permeating from the feed tank to the permeate tank. The higher the concentration of H^+^ ions, the higher the amount of H^+^ permeation through the membrane. The interaction between hydrogen ions and oxygen-containing groups of GO-PHE under high acidity (1 and 2 mol/L nitric acid) results in the protonation of oxygen-containing groups and the reduced electronegativity of GO-PHE [37]. Metal ions can pass through the membrane easily due to the weak electrostatic force with oxygen-containing groups [38].

### 3.4. Permeability of Na^+^ Through GO-PHE-Inserted K^+^, Ca^2+^, and Fe^3+^

To find out the influence of electronegativity of the membrane on metal ion permeation, the GO-PHE membrane was immersed into three kinds of nitric salt solution: KNO_3_, Ca(NO_3_)_2,_ and Fe(NO_3_)_3_ (GO-PHE-K, GO-PHE-Ca, GO-PHE-Fe).

**Figure 8:**
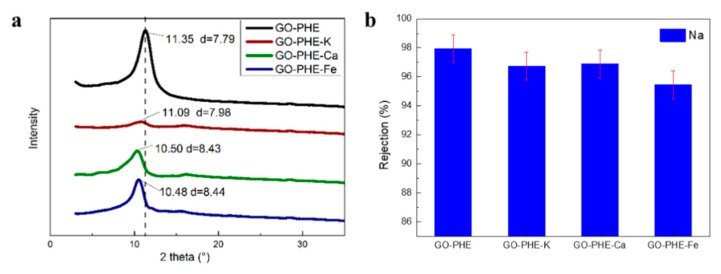
(**a**) XRD spectra of GO-PHE, GO-PHE-K, GO-PHE-Ca and GO-PHE-Fe. (**b**) Rejection coefficient of Na^+^ through GO-PHE, GO-PHE-K, GO-PHE-Ca and GO-PHE-Fe. [NaNO_3_] = 0.05 mol/L, [HNO_3_] = 1.0 mol/L. GO-PHE-K, GO-PHE-Ca, and GO-PHE-Fe was GO-PHE membranes adsorbing KNO_3_, Ca(NO_3_)_2_, Fe(NO_3_)_3_, respectively.

Figure 8a shows the powder diffraction pattern of GO-PHE-K, GO-PHE-Ca, GO-PHE-Fe, and d-spacing values of ~7.98 (GO-PHE-K), ~8.43 (GO-PHE-Ca), and ~8.44 (GO-PHE-Fe), respectively. The d-spacing of GO membranes is enlarged when metal ions enter into the interlayers of GO membranes [35]. Figure S3 shows the K, Ca, and Fe percentage content of GO-PHE-K, GO-PHE-Ca, and GO-PHE-Fe characterized by XPS, respectively. According to the XPS results, the metal ions’ contents are 0.23% (K), 0.71% (Ca), and 0.58% (Fe) in GO-PHE-K, GO-PHE-Ca, and GO-PHE-Fe, respectively. The percentage content of K^+^ is the least within all of the three ions and the effect on the layer spacing is minimal according to Figure 8a. There was almost the same d-spacing of GO-PHE-Ca and GO-PHE-Fe for Fe insertion amount was lower than Ca while Fe has a larger ionic radius.

Figure 8b shows the rejection coefficients of Na^+^ through GO-PHE, GO-PHE-K, GO-PHE-Ca, and GO-PHE-Fe. According to Figure 8b, the rejection coefficient to Na^+^ follows the order GO-PHE > GO-PHE-K ≈ GO-PHE-Ca > GO-PHE-Fe. This is the result of the joint effect of d-spacing and interlaminar metal ions. K^+^ in the GO-PHE-K membrane has the lowest residual amount and therefore the weakest neutralization capacity to the electronegativity in the three kinds of membranes, resulting in the maximal hindrance to Na^+^ permeation. Although the interlayer spacing of GO-PHE-Ca and GO-PHE-Fe are almost the same, different charges of the Ca^2+^ and Fe^3+^ ions make them fairly different in their rejection ability to Na+. According to the residual amount of Ca2+ and Fe3+, we can estimate the neutralization ability of the membranes by simply multiplying the percentage amount of residue ions by their charges, obtaining 0.71 × 2 = 1.42% for GO-PHE-Ca and 0.58% × 3 = 1.74% for GO-PHE-Fe. As a result, GO-PHE-Fe is much more neutralized than GO-PHE-Ca, which facilitates the permeation of Na+ ions and exhibits a lower rejection. The same mechanism also applies for membranes under high acidity, with protonated oxygen-containing groups, the membrane is neutralized by H^+^, thus reducing hindrance to Na^+^ permeation.

### 3.5. Antibacterial Performance of GOM, GO-Ala, GO-PHE, and GO-Ser

**Figure 9:**
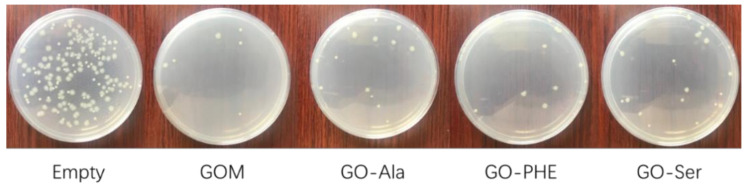
Antibacterial activity of GOMs against *E. coli.*

The bactericidal property is one of the important characteristics of the membranes for maintaining the filtration performance in practical water separation applications [12]. The antibacterial property of the cross-linked GO membrane was evaluated against E. coli using airborne bacteria tests. By counting the colonies of *E. coli*, the antibacterial ability of the membranes can be investigated. As shown in Figure 9, the remarkable difference in the number of colonies between the experimental and control group indicates that large amounts of bacteria decrease with the treatment of GOMs and GOM-A. All of the membranes in our experiment have exhibited antibacterial rates regarding *E. coli* of over 85.3% and exhibit nearly the same antibacterial activity. The antibacterial activity of the membranes was attributed to membrane stress induced by the sharp edges of graphene nanosheets, which may result in physical damages on cell membranes, leading to the loss of bacterial membrane integrity and the leakage of RNA [39]. The membranes are promising for uses in practical water treatments with relatively high antibacterial rates.

## 4. Conclusions

We prepared stable GOM-A (GO-Ala, GO-PHE, GO-Ser) and explored the rejection behavior of metal ions in aqueous solution. The stability of the membranes was improved and the interlayer spacing was adjusted by cross-linking with amino acids. The composite membranes exhibited a high permeation flux (4.72, 6.61, and 11.81 L/m^2^·h) at atmospheric pressure. The rejection coefficient of the GOM-A for monovalent alkali metal ions was over 94%, but was 97% for divalent alkaline earth metal ions. The rejection of metal ions is related to the interlayer spacing of the membrane as well as the number of charges and the hydrated ionic radius of permeating metal ions. Larger interlayer spacing of the membranes and higher acidity induce less rejection to metal ions. Metal ions can be inserted into layers of GOM-A to enlarge the interlayer spacing of GOM-A and neutralize the electronegativity of the membrane. GOMs and GOM-A showed quite good antibacterial properties against E. coli. Our work provides a strategy to improve the stability and water flux of GOMs by cross-linking with amino acid and the produced GOM-A can be suitable to purify and desalt water.

## Figures and Tables

**Table 1:** Contact angle, water flux, stability, and tensile properties study of GOMs.

Membrane	Contact Angle (°)	Water Flux (L m^−2^ h^−1^)	Water Phase * (s)	Tensile Properties (MPa)
GOM GO-Ala	52 44.4	6.07 4.72	14 54	27.2 26.4
GO-PHE	53.8	6.61	36	25.8
GO-Ser	53.2	11.81	43	27.3

Note: * broken time of membranes.

## Supplementary Materials

The following are available online at https://www.mdpi.com/2077-0375/10/10/296/s1, Figure S1: Schematic diagram of measuring water flux. Figure S2: XPS of GOM, GO-Ala, GO-Ser and GO-PHE analyzed by full spectrum. Figure S3: XPS of GO-PHE-K (a), GO-PHE-Ca(b) and GO-PHE-Fe (c). Table S1: Root mean surface roughness (RMS), root average arithmetic roughness (Ra), and root peak-to-valley (Rpv) values of GOMs. Table S2: The permeation flux of metal ions through GO-Ala, GO-Ser and GO-PHE.

## Abbreviations

GO: graphene oxide; GOM: graphene oxide membrane; GOM-A: graphene oxide membranes cross-linked by amino acids; GO-Ala, GO-PHE, GO-Ser: graphene oxide membrane cross-linked by L-alanine, L-phenylalanine, and serine, respectively; GO-PHE-K, GO-PHE-Ca, and GO-PHE-Fe: GO-PHE membranes inserted by K^+^, Ca^2+^, Fe^3+^, respectively; FT-IR: Fourier Transform Infrared Spectrometer; XRD: X-ray diffractometer; SEM: scanning electron microscope; XPS: X-ray photoelectron spectroscopy.
