# Supplementary material for: Amino Acid Cross-Linked Graphene Oxide Membranes for Metal Ions Permeation, Insertion and Antibacterial Properties
Source: Membranes (Basel). 2020 Oct 21;10(10):296. doi: 10.3390/membranes10100296 (PMC7589175; doi:10.3390/membranes10100296)
Supplement: Supplementary file 1 [file membranes-10-00296-s001.pdf]

Supplementary Information

Amino Acid Cross-Linked Graphene Oxide Membranes for Metal Ions Permeation, Insertion and Antibacterial Properties

Lijuan Qian[1,2], Haijing Wang[1], Jingyi Yang[1], Xiaolei Chen[1], Xue Chang[1], Yu Nan[1], Zhuanyan He[1],

Peizhuo Hu[1,2]*, Wangsuo Wu[1,2], Tonghuan Liu[1,2]*

1.School of Nuclear Science and Technology, Lanzhou University, Lanzhou 73000, PR China

2. Key Laboratory of Special Function Materials and Structure Design, Ministry of Education, Lanzhou, Gansu 730000, China

*Corresponding Author: hupzh@lzu.edu.cn, liuth@lzu.edu.cn

1. Preparation of GO

The modified Hummers method was used to prepare GO suspension [21], which was divided into three stages. In the low temperature stage, 5 g of graphite and 2.5 g $NaNO_3$ was added to 110 mL concentrated sulfuric acid at a temperature about 273-278 K successively. Then 20 g $KMnO_4$ was slowly put in the mixture under stirring for 2 h. The second step, 200 mL of deionized water was added and the reaction time lasted about 30 min. In the third step, the temperature was raised to 371K and maintained about 1h. Then H_2O_2 (30%, no more than 10 mL) was slowly added after 200 mL of distilled water was added. The mixture was centrifuged and washed with distilled water until the pH became neutral. The GO concentration was calculated by freeze-drying a certain volume of GO solution and weighed its mass.

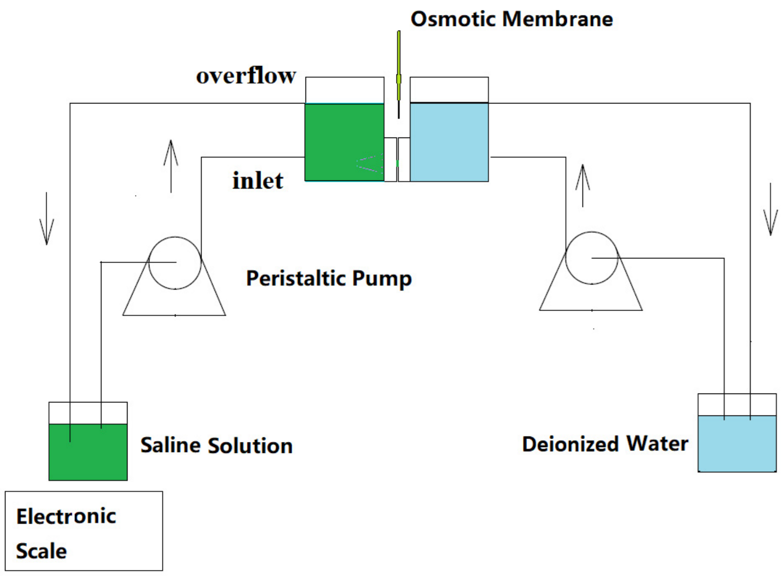

Fig. S1 Schematic diagram of measuring water flux

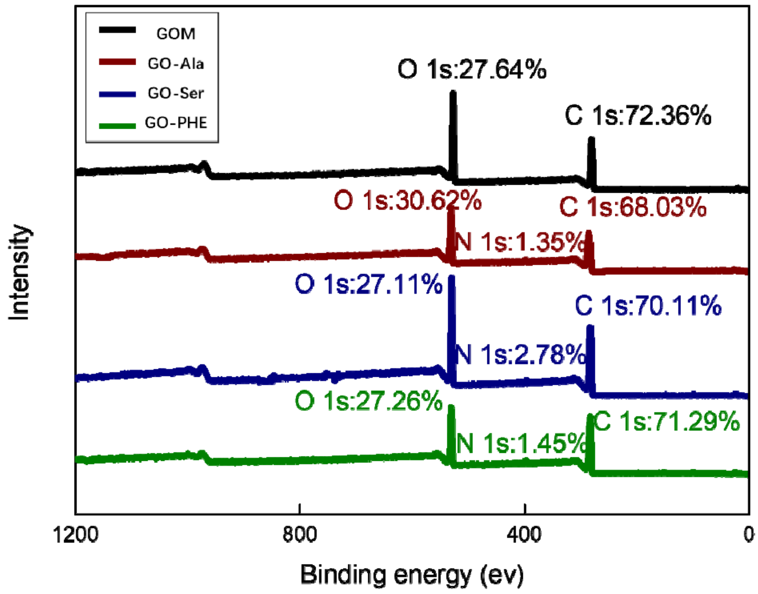

Fig.S2 XPS of GOM, GO-Ala, GO-Ser and GO-PHE analyzed by full spectrum

Table S1 Root mean surface roughness (RMS), root average arithmetic roughness (Ra), and root peak-to-valley (Rpv) values of GOMs

Membrane	Roughness(nm)		
	R_{max}	R_a	R_{pv}
GOM	246	37.8	395
GO-Ala	173	25.7	289
GO-PHE	258	31.3	432
GO-Ser	195	28.4	343

Table S2 The permeation flux of metal ions through GO-Ala, GO-Ser and GO-PHE

Ion species	Permeation flux (mmol h^{-1} m^{-2})		
	GO-Ala	GO-PHE	GO-Ser
Na^+	0.038	0.039	0.039
K^+	0.043	0.045	0.044
Cs^+	0.039	0.041	0.040
Mg^{2+}	0.011	0.015	0.013
Ca^{2+}	0.018	0.022	0.018
Sr^{2+}	0.014	0.017	0.015

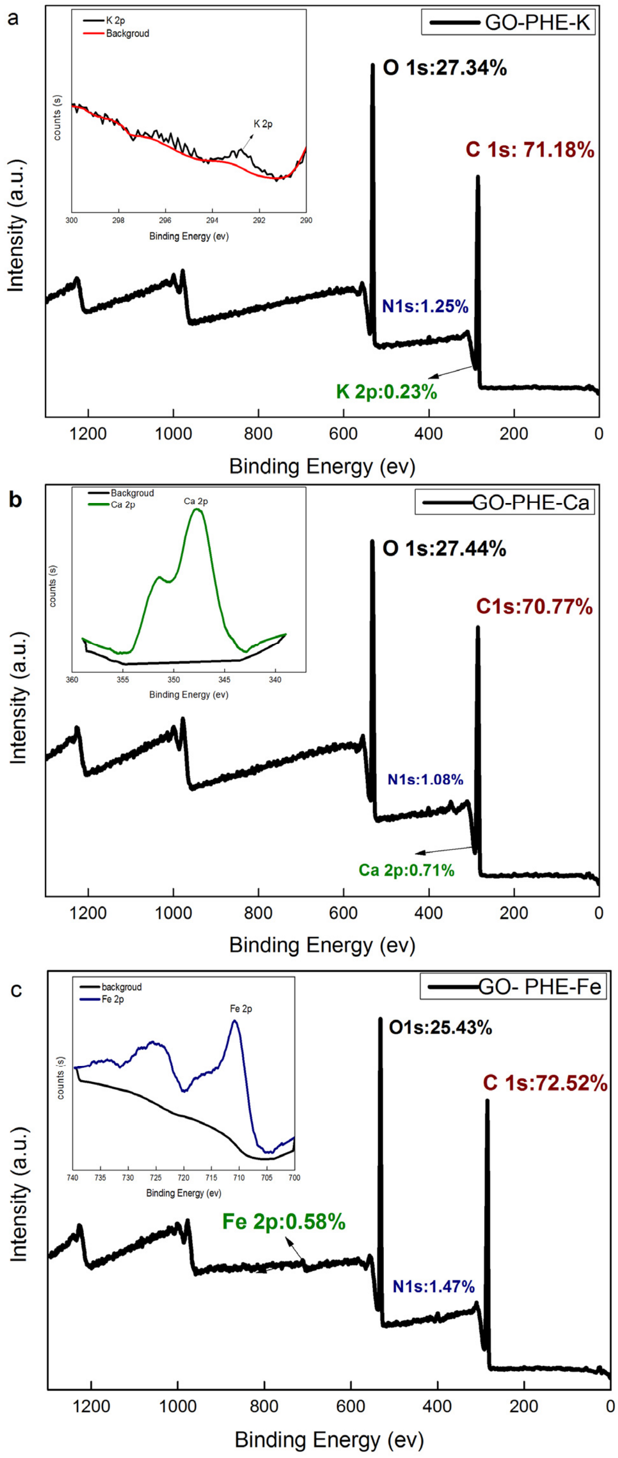

Fig.S3 XPS of GO-PHE-K (a), GO-PHE-Ca(b) and GO-PHE-Fe (c)
